# CAS-SFCM: Content-Aware Image Smoothing Based on Fuzzy Clustering with Spatial Information

**DOI:** 10.3390/jimaging11060173

**Published:** 2025-05-22

**Authors:** Felipe Antunes-Santos, Carlos Lopez-Molina, Maite Mendioroz, Bernard De Baets

**Affiliations:** 1 Department of Statistics, Computer Science and Mathematics, Public University of Navarre (UPNA), 31006 Pamplona, Spain; 2 IdiSNA, Navarra Institute for Health Research, NavarraBiomed, Hospital Universitario de Navarra, 31008 Pamplona, Spain; tmendioi@navarra.es; 3 KERMIT, Department of Data Analysis and Mathematical Modelling, Ghent University, 9000 Ghent, Belgium; bernard.debaets@ugent.be

**Keywords:** image processing, image smoothing, content-awareness, fuzzy clustering

## Abstract

Image smoothing is a low-level image processing task mainly aimed at homogenizing an image, mitigating noise, or improving the visibility of certain image areas. There exist two main strategies for image smoothing. The first strategy is content-unaware image smoothing. This strategy replicates identical smoothing behavior at every region in the image, hence ignoring any local or semi-local properties of the image. The second strategy is content-aware image smoothing, which takes into account the local properties of the image in order to adapt the smoothing behavior. Such adaptation to local image conditions is intended to avoid the blurring of relevant structures (such as ridges, edges, and blobs) in the image. While the former strategy was ubiquitous in the early years of image processing, the last 20 years have seen an ever-increasing use of the latter, fueled by a combination of greater computational capability and more refined mathematical models. In this work, we propose a novel content-aware image smoothing method based on soft (fuzzy) clustering. Our proposal capitalizes on the strengths of soft clustering to produce content-aware smoothing and allows for the direct configuration of the most relevant parameters for the task: the number of distinctive regions in the image and the relative relevance of spatial and tonal information in the smoothing. The proposed method is put to the test on both artificial and real-world images, combining both qualitative and quantitative analyses. We also propose the use of a local homogeneity measure for the quantitative analysis of image smoothing results. We show that the proposed method is not sensitive to centroid initialization and can be used for both artificial and real-world images.

## 1. Introduction

Smoothing has become a key part of different computer vision procedures over the past decades. This task is not necessarily related to denoising; rather, it seeks to simplify and regularize the image prior to further image analysis. Originally, the predominant smoothing strategies were based on Gaussian kernels, especially after they were proven to be the only kernels not producing new maxima or minima in the first derivative of the signal [1,2]. Gaussian kernels were, indeed, a fertile field of study, which led to developments such as the Gaussian Scale-Space [3,4] and anisotropic Gaussian kernels [5,6,7]. However, it soon became clear that, while Gaussian kernels were adequate for signal regularization and simplification, this came at the cost of blurring object boundaries and even removing small visible structures of the image. The concern over these pernicious effects led to the study of content-aware smoothing techniques, which could eventually combine signal regularization within the relevant objects in a given image with preservation (or even sharpening) of the object boundaries.

Content-aware smoothing (CAS) techniques have been studied for more than 30 years, combining different inspirations and embodiments. In most cases, CAS is aimed at intra-region color regularization with inter-region difference preservation or sharpening. Initial attempts were based on local, discrete schemes that avoided inter-region edge blurring using local cues. In this regard, we can find pioneering efforts specifically mentioning these goals, such as the work by Newman and Dirilten on strip-mask transformations [8]. A more advanced proposal was made by Saint-Marc et al., firstly presenting adaptive kernels for image regularization [9] and further expanding their proposal in [10]. This was also a pioneering effort to create non-isotropic scale-spaces, as opposed to the isotropic scale-spaces (mostly, the Gaussian Scale-Space), which were gaining traction in preceding years [11]. With goals similar to those of Saint-Marc et al., Perona and Malik took inspiration from the heat diffusion process to propose an original iterative, discrete schema for *anisotropic diffusion* [12], which results in a non-isotropic scale-space. Over the years, other methods have inspired a wide range of people to realize CAS. Two main trends can be distinguished in these efforts. On the one hand, some methods were based on local image analysis, attempting to predict the presence of boundaries in the image locally so as to adapt the local smoothing behavior. Examples of this trend are the discrete schemata in [9,10,12], but also the continuous schemata based on *anisotropic diffusion* [13] or bilateral filtering [14]. On the other hand, some authors took a data-oriented interpretation of the image, which allowed for the iterative analysis (often, clustering) of the pixels in the spatio-tonal universe. Comaniciou and Meer [15], for example, presented Mean-Shift, a procedure for image smoothing based on a global clustering of all pixels, resulting in progressive CAS of the image. Similar goals have been achieved by, for example, gravitational clustering [16]. While some of these alternatives have been proven to be fundamentally equivalent [17,18], their interpretation is different: the former relies on local boundary cues, whereas the latter relies on the global pixel distribution in the spatio-tonal space.

Smoothing, in general, and CAS in particular, have a wide range of applications. It serves mostly for the pre-processing of images prior to their automated analysis, but it can also be used, for example, to simplify the visual inspection of images. Works presenting direct applications of CAS can be found in ultrasound imaging [19], photogrammetry [20], and microscopy [21,22]. Moreover, some studies do not focus on the type of image but on the analysis technique to be supported by CAS, e.g., segmentation [23] or image compression [24].

This work presents a clustering-based CAS technique rooted in fuzzy clustering. Our proposal is data-oriented and is based upon the hypothesis that different visual regions should be numerically separable in the spatio-tonal universe. Our proposal also capitalizes on the inner workings of soft clustering methods in order to inspect and process spatio-tonal information. Specifically, we use Spatial Fuzzy *c*-Means (SFCMs), a variant of the popular Fuzzy *c*-Means, as the fuzzy clustering framework for our proposal. SFCMs embodies the desired behavior for the soft clustering of the datasets created from given images. This article is a revised and expanded version of a paper entitled *Content-Aware Image Smoothing Based on Fuzzy Clustering*, which was presented at the International Conference on Information Processing and Management of Uncertainty in Knowledge-Based Systems (IPMU), held at Milano-Bicocca (Italy) [25].

The remainder of this paper is organized as follows. Section 2 presents the intuition of using clustering methods to perform image smoothing, and Section 3 presents SFCMs in particular, how it can be used to perform image smoothing, and our new proposal. Section 4 and Section 5 present the qualitative and quantitative approaches taken to evaluate our proposal, respectively. Lastly, Section 6 presents the conclusions of our work.

## 2. Clustering-Based Image Smoothing

Among the inspirations for content-aware smoothing (CAS), a relevant trend is that inspired by multidimensional clustering. This trend is based on the interpretation of images as datasets. In this interpretation, pixels become instances to be analyzed from a spatio-tonal perspective. Let I:Ω↦T be an image, with Ω⊂N×N representing the set of positions and T representing the tonal palette. In order to perform spatio-tonal clustering on the pixels of *I*, each pixel becomes an instance $p\in R^{+}\times R^{+}\times T$. For a pixel I(x,y)=t∈T with tone $t=(t_{1},\ldots ,t_{k})$, its representation in the spatio-tonal universe will be $\left(x,y,t_{1},\ldots ,t_{k}\right)$.

The foundational idea of clustering-based CAS is that clustering methods shall group together pixels that are both spatially and tonally similar. This leads to intra-region color regularization. Additionally, pixels that are spatially similar but tonally different must not be grouped together, preventing inter-region edge blurring. While these ideas appear natural, the application of clustering methods to CAS is not direct. The final stage of a clustering process produces a partition of the pixels into classes, which is a segmentation (not a smoother image). Hence, in order to perform CAS using a clustering method, it is necessary to exploit the information generated during the clustering process, not just at its end. In other words, it is necessary to design a procedure that is able to produce a progressively smoother image from the information in the clustering process.

There is no generic approach to produce smoother images using a clustering method. Instead, it is a process that is rather dependent upon the specific clustering method used. Assuming the clustering method is iterative, the goal is to build an image at each iteration of the process. The generation of images at each stage of the clustering process is, again, heavily dependent upon the specific information generated by the process itself. In this regard, we can discriminate between the two main types of clustering methods.

In clustering methods that properly evolve (modify) the instances in the dataset (e.g., Mean-Shift [15] or gravitational clustering [26]), each instance in the image will be modified iteratively, affecting both the tonal and the spatial information. Interestingly, the spatial information will no longer fit the original pixel grid in Ω. Hence, when using such a clustering method, positional information is normally reset back to the original values after each iteration in the clustering process, and tonal information is progressively modified. In this way, one image can be built at each iteration by resetting the spatial information in the instances and rebuilding an image with the updated tonal information.

In clustering methods that evolve a set of cluster centroids, leaving the instances unaltered, the situation is rather different. In such cases, it is necessary to elaborate on the information at each cluster centroid, as well as on the relationship between the cluster and the instances. Different scenarios can be found if the membership instance-to-cluster is expressed as a probability, a degree of membership, or any other numerical representation. Anyhow, since each centroid is an element of $R^{+}\times R^{+}\times T$, it does represent a tone. Hence, at each iteration of the clustering process, an image can be reconstructed using a combination of the tones of the centroids, using, at each pixel, the *memberships* to such clusters as weighing or modulating factors. Note that this cannot be carried out if the assignment of instances to clusters is crisp (binary), hence our interest in using fuzzy clustering, which assigns to any given image a partial membership to each cluster. While the specific combination is dependent on the numerical nature of both the cluster centroid and the instance-to-cluster *memberships*, all solutions are fundamentally similar. For example, if a pixel undoubtedly belongs to a specific cluster at some iteration, the color of the pixel at such iteration will be exactly that of the cluster centroid. To the best of our knowledge, other types of clustering methods, such as agglomerative clustering methods, have not been applied in the context of image smoothing.

Apart from creating images at each iteration of the clustering process, a key technical issue in clustering-based CAS is the design of comparison measures (often, metrics) for the spatio-tonal universe. This should, for example, take into account the variability in the potential tonal palette T, which can range from grayscale tones (scalar values in $R^{+}$ or $N^{+}$) to hyperspectral signatures (vectors in ${\left(R^{+}\right)}^{256}$ or ${\left(R^{+}\right)}^{512}$), as well as the fact that spatial and tonal information might come in very different scales.

It is standard practice to produce a metric through the convex combination of two metrics: one in the spatial universe (Ω) to account for spatial similarity, and another one in the tonal universe (T) to account for the tonal similarity. Incorporating (numerically) each universe in the convex combination demands careful adjustment to ensure the representativeness of both the spatial and tonal information in the spatio-tonal metric. Notably, the design of comparison measures able to quantify distances in a spatio-tonal universe is recurrent in the image processing literature. For example, it was a key in the evolution of Baddeley’s delta metric [27] from binary images to grayscale images [28].

It is relevant to recall that a clustering method, at its final stage, can also be used for both segmentation and hierarchical segmentation. The literature contains different successful examples, such as the graph-based hierarchical clustering method by Felzebschwalb and Huttenlocher [29] or the FCM-based segmentation by Yang et al. [30]. In clustering-based segmentation procedures, there is no need to produce intermediate images as the clustering evolves since the only required result is the partition of the spatial universe. However, they do require spatio-tonal comparison measures for the clustering and, hence, can be used as inspiration for clustering-based CAS.

## 3. Content-Aware Image Smoothing Using Spatial Fuzzy c-Means

Fuzzy *c*-Means (FCMs) is a fuzzy clustering method with roots in the works by Dunn [31] and Bezdek et al. [32,33]. Presented as a generalization of the *k*-Means algorithm, FCMs is built around a soft representation of the instance-to-cluster membership. In FCMs, clusters are represented as membership functions, each associated with a cluster centroid, which jointly make up a Ruspini partition. The algorithm has been extensively used since its introduction, and contributions can be found both in evolving the mathematical model and adapting it to specific fields of application [34,35,36]. Among these variants of FCMs, a very relevant one is Spatial Fuzzy *c*-Means (SFCMs), which focuses on the generalization of the metric used to compute the membership functions and, hence, the membership of each instance to the clusters [37]. We capitalize on SFCMs to perform content-aware image smoothing. To better explain our proposal, we present SFCMs in Section 3.1. Section 3.2 reviews the most relevant aspects of clustering-based CAS, leading to a detailed description of our proposal (Section 3.3).

### 3.1. Spatial Fuzzy c-Means

Spatial Fuzzy *c*-Means (SFCMs) was presented by Chuang et al. [37] in 2006 as an unsupervised clustering method aimed at image segmentation. They argue that FCMs, in its standard version, overlooks the spatial information component of images, specifically ignoring the fact that neighboring pixels in images are highly correlated [37].

Explicitly, SFCMs takes as input an image *I*, with I:Ω↦T, where Ω={1,…,M}×{1,…,N} represents the set of positions in the image, and T is a color palette. Both SFCMs and FCMs produce a Ruspini partition of the universe Ω×T using a fixed number of clusters *r*, each of which is represented by a membership function $\mu_{i}:\Omega \times T\mapsto \left[0,1\right]$, i=1,…,r. In order to do so, at each iteration *t* of the clustering process, the algorithm maintains a list of *r* cluster centroids $C_{t}=\left(C_{t,1},C_{t,2},C_{t,3},\ldots ,C_{t,r}\right)$, with $C_{t,i}\in \Omega \times T$. Both the fuzzy partition (in terms of membership functions) and the update of the cluster centroids are computed using a distance metric on the universe Ω×T, which is, hence, required to quantify closeness and farness in the spatio-tonal universe.

### 3.2. Adapting SFCMs to Image Smoothing

While SFCMs was originally aimed at image segmentation [37], its soft representation of data can be used as a cornerstone for a CAS procedure [25]. There are four main aspects in soft clustering methods that require attention in order to perform a proper image smoothing. These main aspects are the following: (a) the number of clusters used in the procedure, (b) the cluster centroid initialization, (c) the metric used to compute distances in the spatio-tonal universe, and (d) the reconstruction of smooth images from the clustering output. We explore each aspect individually.

(a) The number of clusters in a clustering method represents the number of distinct classes that the data are to be divided into [38], as each cluster is supposed to be a group of similar instances within the dataset [39]. In terms of image processing, the classes represent distinct regions within an image [37]. There are multiple strategies to set the number of clusters in clustering methods [39]. One strategy is to consider the number of clusters as a manual parameter that is to be set by a human operator [32,39,40]. Another strategy is to consider the number of clusters as a parameter to be optimized and to use a clustering quality measure (e.g., the silhouette coefficient) for this purpose [41,42]. Yet another strategy is to consider the number of clusters as a parameter to be extracted from the data distribution [43]. The frequencies of the tones within the dataset are mapped, and then the peak frequencies are analyzed to estimate the number of clusters within the data, much like the histogram thresholding method presented by Otsu in 1979 [43,44].

(b) Centroid initialization refers to determining the position of the cluster centroids in clustering methods [45,46]. Different such initializations might result in different clustering outputs [47], an effect often referred to as *initialization sensitivity* [48]. There exist various techniques for centroid initialization of clustering methods [45]. A common approach is to select *n* random instances from the dataset and use them as initial cluster centroids. This approach is referred to as Forgy initialization [46]. Given the extreme variability caused by such a randomized process, Arthur and Vassilvitskii presented k-means^++^, an alternative initialization aiming at mitigating initialization sensitivity [47]. In k-means^++^, the first cluster centroid is selected randomly from the dataset. The second centroid is then selected as the instance in the dataset that is located farthest from the first centroid. The process is then repeated until all cluster centroids have been selected, always selecting the instance located farthest from the already selected centroids. A third initialization method, which does not properly address the issue of initialization sensitivity, is random partition initialization [45,47]. In this approach, instances are randomly assigned to one of *c* clusters.

(c) In the present context, metrics have the role of quantifying the closeness and farness of the instances to the centroids [39]. The definition of a sensible metric is very relevant in clustering-based CAS since it is required to yield values according to human interpretation of images. Ideally, the metric must yield low values in the comparison of centroids to the pixels that belong to the region represented by such a centroid and high values when the comparison involves a centroid and the pixels not belonging to the object it represents. This is not straightforward since different objects or regions in an image can have highly variable spatio-tonal characteristics. For example, when characterizing a large background, spatial information is of little or no use. However, in small, textured objects, the spatial information might be the key to successful clustering.

**Figure 1:**
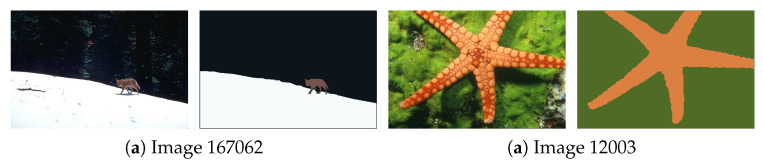
Sample images from BSDS500 [49], together with a visual representation of their distinct regions. The regions are extracted from the human-labelled ground truth in BSDS500.

**Figure 2:**
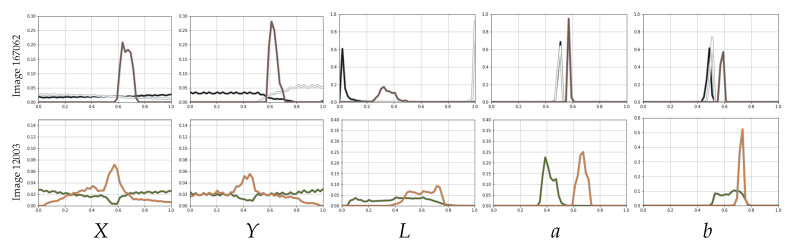
Distribution of the feature values from the images in Figure 1 in each dimension of the spatio-tonal universe. *X* and *Y* refer to the horizontal and vertical position of the pixels, and *L*, *a*, and *b* are the CIELab color components. Each distribution takes the color of the distinct region it represents, as depicted in Figure 1. The distributions are normalized for each distinct region.

Figure 1 contains two images from BSDS500 [49], featuring two to three distinct regions, and Figure 2 displays the per-class frequency plots for each dimension once projected into the spatio-tonal universe (using the CIELab color space). In this figure, we can observe how different regions might have very different representations in different dimensions. For example, the wolf in Image 167062 has a very strong spatial coherence, but both foreground and background regions would be better characterized exclusively in terms of color, mostly in terms of luminance *L*. The images in Figure 1 illustrate that the spatial and tonal information can have different relevance to each object or region. Hence, a suitable metric should be prepared to modulate the relevance of spatial and tonal information.

(d) The reconstruction of images from the clustering information is highly dependent on the information generated by the clustering method. Within SFCM, as well as within the original FCMs, cluster centroids are used to create a Ruspini partition [50] of the clustering space, which, in this case, is the spatio-tonal universe Ω×T. When provided with such a partition, in terms of membership functions, each element in the universe will have a membership to each cluster. As the centroids evolve in the clustering process, such memberships will adapt while still conforming to a Ruspini partition. Ideally, each cluster will represent a distinct region within the image. Thus, the *membership degrees* can be understood as the confidence in the membership of the pixel in each of the clusters. In the case of full certainty, with the pixel having membership 1 to some cluster, the pixel shall take the color of the cluster centroid. However, in cases where a pixel has non-zero memberships to two or more clusters, the (tonal) information from their centroids must be combined to determine the color of the pixel. In this work, we intend to use the membership degrees to the different clusters as weights in a convex combination of the tonal information of the cluster centroids. While we fully understand that membership degrees are not weights, we elaborate on their numerical representation to combine the information from different clusters.

### 3.3. A Proposal for Content-Aware Image Smoothing Using SFCMs

In this section, we present a detailed description of CAS-SFCMs based on the analysis in the previous sections. CAS-SFCMs is a content-aware image smoothing algorithm that produces a sequence of progressively smoother images, ending in a non-trivial state (unlike other alternatives such as Mean-Shift and Anisotropic Diffusion).

In CAS-SFCMs, the number of clusters is a parameter to be set by the user and shall reflect the number of distinct regions within the image. An automated selection of the number of clusters would most likely lead to an increase in the complexity of the method. Thus, manual selection is chosen in order to keep CAS-SFCMs as simple as possible without hindering its potential for generalization. The clustering is started using Forgy initialization since the benefits of more intricate initialization methods are unclear.

Regarding the metric in the spatio-tonal universe, CAS-SFCMs uses a convex combination of two metrics, $d_{\omega }$ and $d_{\tau }$, which apply to the spatial and tonal domains, respectively. In general, any metric ${\left(\Omega \times T\right)}^{2}\mapsto R^{+}$ could be used to compute the distance between instances and centroids during the clustering procedure. However, the decomposition into two individual metrics allows for a more delicate understanding of the spatial and tonal information in the image. Once the metrics are defined, we propose to fuse their output through a convex combination:

(1) $$d_{\psi }\left(a,b\right)=\alpha d_{\tau }\left(\hat{a},\hat{b}\right)+\left(1-\alpha \right)d_{\omega }\left(\tilde{a},\tilde{b}\right)\;,$$

where α∈[0,1], a,b∈Ω×T, and $\hat{a},\hat{b}\in T$ (resp. $\tilde{a},\tilde{b}\in \Omega$) are the tonal (resp. spatial) information in *a* and *b*. We recommend setting $d_{\omega }$ to the Euclidean metric and adapting $d_{\tau }$ to the specific color space used in the imagery to be processed. In this work, since our examples are restricted to the CIELab color space, we also set $d_{\tau }$ to the Euclidean metric [51]. The spatio-tonal parameter α controls the relative contributions of the spatial and tonal information in the image smoothing. Setting α=0 would totally ignore tonal information, hence rendering the algorithm as CUS. Alternatively, setting α=1 would cluster tonal information regardless of the spatial structure of the visual information. Moreover, α should be adjusted to the actual tonal and spatial compactness of the objects in the image, with prevalence given to the components that actually characterize each object in the spatio-tonal universe. Qualitative experiments illustrating the impact of this parameter are provided in the upcoming sections.

CAS-SFCMs produces an image at each iteration of the clustering process, progressively smoothing the original image. At each iteration, the image is produced by combining information about the clusters that each pixel (partially or totally) belongs to. Let $\mu_{t,i}:\Omega \mapsto \left[0,1\right]$ be the membership function of the *i*-th cluster at the *t*-th iteration. The membership degree of any pixel *p* to this cluster is given by $\mu_{t,i}\left(p\right)$. The value of each pixel in the image $I_{t}$ is given by

(2) $$I_{t}\left(x,y\right)=\sum_{i=1}^{r}\mu_{t,i}\left(q\right)\cdot {\hat{C}}_{t,i}\;,$$

where ${\hat{C}}_{t,i}$ represents the tonal information of the *i*-th cluster centroid at the *t*-th iteration, *r* is the number of clusters, and q∈Ω×T represents the pixel at position (x,y)∈Ω.

**Figure 3:**
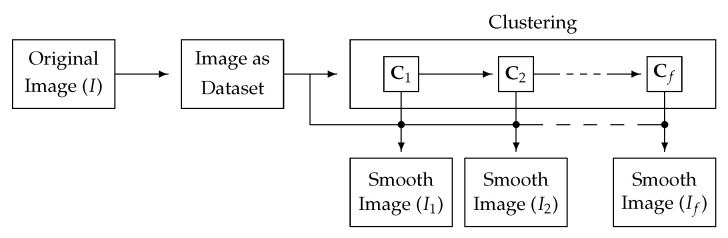
Schematic representation of CAS-SFCMs. The image *I* is mapped into a dataset. Then, the clustering procedure iteratively produces cluster centroids. Hence, the information from the cluster centroids from each iteration can be used to generate a progressively smoother image, as covered in Section 3.3.

Our proposal is schematically depicted in Figure 3 and described in Algorithm 1. As shown in the algorithm, the clustering method will produce a set of cluster centroids $C_{t}$ to be updated at each iteration. At each iteration, we use the information of the cluster centroids, together with that in the original dataset, to produce a smoother image. In the remainder of this work, the images $I_{t}$ are subjected to progressive CAS as *t* increases. Note that the stopping condition of the process is controlled by computing the difference v between consecutive images, hence ending in a non-trivial state (as opposed to other CAS procedures such as bilateral filtering or Mean-Shift). The *difference* Φ(A,B) between any two images (*A* and *B*) is given by

(3) $$\Phi \left(A,B\right)=\frac{1}{\left|\Omega \right|}\sum_{(x,y)\in \Omega }d_{\tau }\left(A\left(x,y\right),B\left(x,y\right)\right)\;.$$

**Algorithm 1** CAS-SFCMs**Parameters**: An image *I*, number of distinct regions *r*, spatio-tonal parameter α and tolerance δMap *I* into a dataset DDefine *r* initial cluster centroids, creating $C_{0}=\left\{c_{1,0},c_{2,0},\ldots ,c_{r,0}\right\}$Compute the initial membership functions $M_{0}=\left\{\mu_{1,0},\mu_{2,0},\ldots ,\mu_{r,0}\right\}$ using $C_{0}$Generate $I_{0}$ from $C_{0}$ and $M_{0}$ using Equation (2)Initialize v←∞While v>δ    Update $C_{t}$ using Equation (1)    Update $M_{t}$ from $C_{t}$    Generate $I_{t}$ from $C_{t}$ and $M_{t}$ using Equation (2)    Update $v\leftarrow \Phi (I_{t},I_{t-1})$ using Equation (3)

While oversimplistic for other tasks, this approach is transparent and meaningful enough for our purposes.

Our proposal is coined as content-aware smoothing using Spatial Fuzzy *c*-Means (CAS-SFCMs). An implementation of CAS-SFCMs is available upon contacting the corresponding author. The upcoming sections are devoted to the study of the different factors of interest for their configuration and use.

In terms of computing time, CAS-SFCMs is relatively light. It was implemented in Matlab2025 for performance analysis, with no parallelization or low-level (MEX) optimization. In a standard PC (Ryzen7, 32 GB) and using double precision floating point numbers, time per iteration ranges from 0.06 s (images in the BSDS500, with 0.3 MPixels) to 1.90 s (images with 5 MPixels). Since CAS-SFCM can be fully implemented in terms of matrix operations, a range of optimization techniques are available depending on the conditions of the machine to be used.

### 3.4. Comparison to Other Alternatives in the Literature

Our proposal is connected to many other alternatives for CAS in the literature. However, the decision to choose an algorithm based on fuzzy clustering is based on a number of considerations. Specifically, our proposal rests on three pillars: avoidance of local image interpretation, centroid-based clustering, and fuzzy interpretation of the partial memberships of instances to clusters.

Firstly, we intended to avoid the use of local information, which can lead to misinterpretations in textures, smooth tonal changes, and visual imperfections. Local feature-based algorithms (such as Anisotropic Diffusion) use gradients or tensors because their goal is to prevent diffusion across object boundaries [13] or to increase it along linear structures [52]. In our case, clustering is preferred because we intend to perform a global (image-level) interpretation of pixel distributions in order to preserve visually salient regions.

Secondly, the use of centroid-based clustering relates to the need to produce convergent, non-trivial results. With respect to non-trivial convergence, CAS algorithms can be classified into two main categories. Some classical alternatives for CAS, such as Anisotropic Diffusion [12,13], progressively smooth the image until a final trivial state is reached, which usually corresponds to a flat image in which all pixels contain the average tone in the image. This is carried out because, while dependent upon local conditions of the image, smoothing is never null [53]. This is also the case of CAS algorithms based on centroid-less clustering, that is, agglomerative clustering algorithms. For example, CAS, based on gravitational clustering, considers each pixel as a celestial body in a 5D space. These instances are iteratively clustered by simulating the gravitational forces between them [16]. As pointed out in the introduction, the spatial coordinates are reset to their original values after each iteration. Still, the color components of each 5D instance tend to homogenize, finally converging into a single tone. Since gravitational forces are (exclusively) attractive and non-zero, the system cannot converge to any state other than the trivial one. The only algorithms allowing for non-trivial convergence are centroid-based, which, in turn, require the setting of a number of centroids.

Lastly, the interest in using fuzzy clustering relates to the generation of intermediate images at each iteration of the process. When using crisp (non-fuzzy) clustering, as in *k*-means, each instance belongs exclusively to one cluster. In such conditions, an intermediate image could only be composed of as many tones as there are clusters in the image, with each pixel representing the tone of its assigned cluster. Moreover, reassigning pixels between clusters at one given iteration would make pixels change color abruptly at such an iteration, producing a sense of instability that does not match the progressive smoothing effect expected in CAS. Fuzzy clustering allows for the weighted combination of the tones of all clusters at each pixel.

## 4. Qualitative Analysis of CAS-SFCMs

This section is devoted to studying how CAS-SFCMs can be effectively used in different scenarios. Specifically, we explore the impact of the number of clusters (Section 4.1), the cluster centroid initialization (Section 4.2), and the spatio-tonal parameter α (Section 4.3).

### 4.1. Number of Clusters

**Figure 4:**
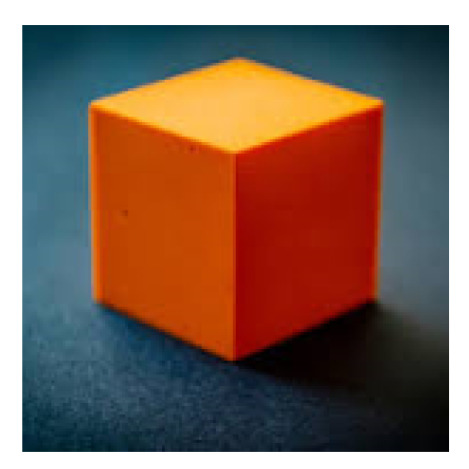
Synthetic image used to evaluate the role of the number of centroids in CAS-SFCMs. Depending on the interpretation of the image, it contains from two to five distinct regions.

**Figure 5:**
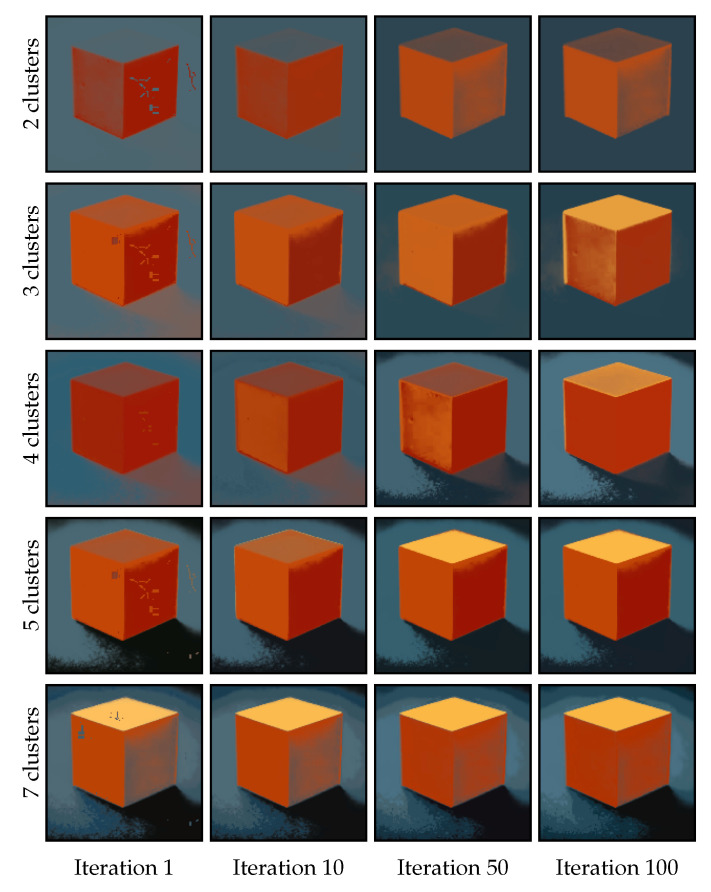
Results obtained by CAS-SFCMs on the image in Figure 4 using a different number of clusters *r* after 1, 10, 50, and 100 iterations. The cluster centroids are initialized using Forgy initialization, α=1, and both $d_{\omega }$ and $d_{\tau }$ are set to the Euclidean metric. We observe how the number of clusters has a straightforward impact on the results, with the process actually identifying the *r* most distinct regions in the image for each *r*.

The number of clusters in our proposal is a parameter and should correspond to the number of distinct regions in the image in order to achieve optimal smoothing results. We used the image in Figure 4 to analyze the impact of an optimal or non-optimal number of clusters on the smoothing procedure. Figure 4 has five distinct regions, although it could be argued that it contains two (cube and background) or four (each side of the cube, plus the background). Hence, it is interesting to observe how the number of regions leads to a different interpretation of the image. Figure 5 presents the results obtained by applying CAS-SFCMs to the image in Figure 4 with a fixed number of iterations, using two, three, five, and seven as the number of clusters. Note that the cluster centroids are initialized using Forgy initialization.

When inspecting the results in Figure 5, we can draw two distinct conclusions. Firstly, the number of clusters, indeed, has a critical impact on the process. Secondly, we find that the behavior of CAS-SFCMs is predictable when the number of clusters does not match the number of distinct regions in the image. We observe critical differences in the results for each row in Figure 5, with all of them explainable on the basis of the number of centroids. Specifically, the color of each region tends to adapt to the underestimation or overestimation of the number of clusters in a predictable manner. For example, in the upper row of Figure 5, for r=2, we observe how all blueish regions take a dark-blue tone, whereas all orange regions homogenize to a combined mid-orange tone. In addition, for r=7, the existing regions are divided into sub-regions with multiple color tones, as seen in the sides of the cube. It is also relevant to note that the number of regions, as interpreted by a human, might be misleading. For example, when increasing from r=3 to r=4, we observe that a fourth region is created in the background, not in the sides of the cube, as a human might have predicted. This is due to the numerical reality of images, and it can be solved by using spatio-tonal metrics which adapt to the interpretation of the images and their context.

Overall, from Figure 5, we see that the algorithm succeeds in identifying as many distinct regions as the given number of clusters. Additionally, we see that the colors taken by the pixels are predictable and aligned with the number of clusters.

### 4.2. Centroid Initialization

In CAS-SFCMs, centroid initialization should have little or no impact on the output. Unlike the number of clusters, which can be set by visually analyzing the image, determining appropriate initial cluster centroids is far from evident. Our hypothesis is that, whichever random initialization is used, the clustering process is robust enough to make the randomness in the Forgy initialization irrelevant after a certain number of iterations. Otherwise said, the clustering process is expected to converge to very similar results when starting from different cluster centroid initializations.

**Figure 6:**
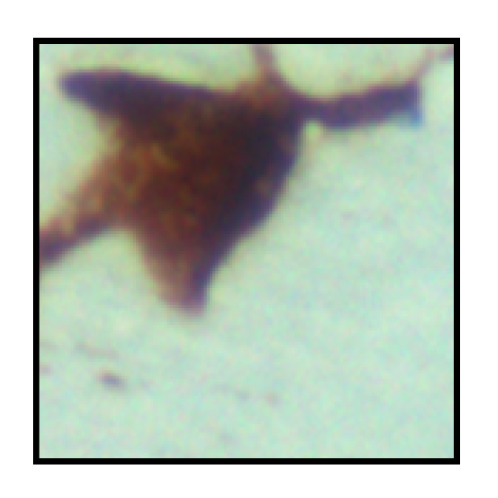
Image patch from an immuno-histochemical sample of human neural tissue. The image has two distinct regions: the light blue cytoplasm from the neural tissue and the brownish tau protein deposit highlighted by an immuno-histochemical staining process.

In order to illustrate this, we used CAS-SFCMs to smooth the image in Figure 6 using 30 different Forgy initializations. In this way, we aimed to verify how sensitive CAS-SFCMs is to randomness in the cluster centroid initialization. In this experiment, we computed, at each iteration, the difference for every pair of images in the pool ($\frac{30\cdot 29}{2}$ pairs per iteration). The *difference* between two images was quantified using Equation (3), i.e., the average pixel-to-pixel tonal distance (in the CIELab color space).

**Figure 7:**
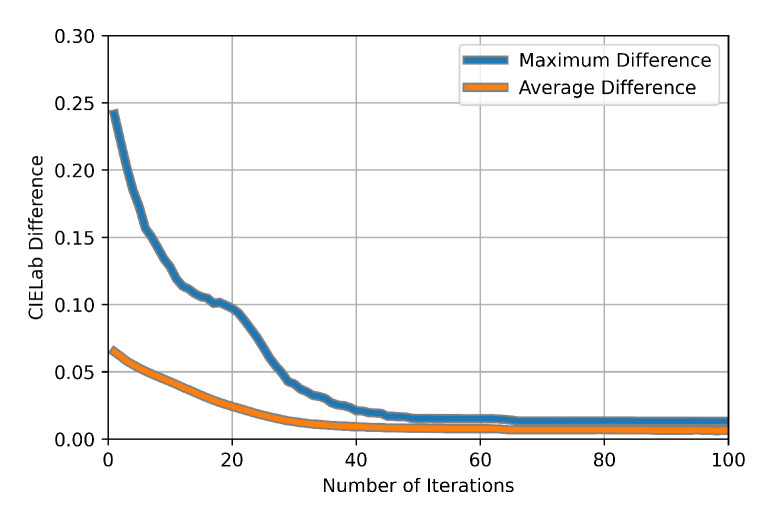
Plot representing the maximum and average differences for all pairs of images in 30 random initializations of CAS-SFCMs applied to the image in Figure 6. The differences were computed at each given iteration using Equation (3). The initialization was performed using Forgy initialization, with the number of clusters at r=2 and α=1.0.

Figure 7 displays, at each iteration, the maximum and average differences for all pairs of images resulting from the 30 centroid initializations. In this figure, we observe that the random nature of the initialization can lead to large discrepancies in the initial steps of the clustering process. However, after some iterations, such discrepancies are drastically reduced, both in terms of maximum and average image differences. Although the differences do stabilize at very low values, they are not reduced to zero. This means that the random initialization has an impact on the results, although it is minimal and tends to be reduced as the number of iterations increases. Otherwise said, the initialization might impact the results in the early iterations of CAS-SFCMs, but it will have a rather light impact as the number of iterations increases. In this sense, Figure 7 shows that CAS-SFCMs is inherently robust to the random nature of the cluster centroid initialization.

### 4.3. Spatio-Tonal Parameter

**Figure 8:**
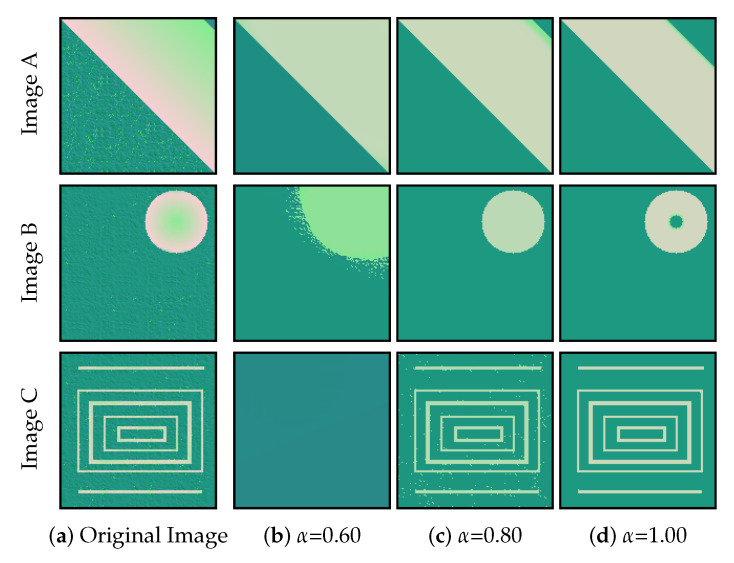
Experiment illustrating the impact that the spatio-tonal parameter α has on the smoothing output. The left-most column contains the original images for the experiment. The three right-most columns contain the results after 100 iterations using different values of the spatio-tonal parameter α.

Understanding the spatio-tonal weight is relatively straightforward. However, the effect it has on the image smoothing procedure needs to be closely analyzed. In order to do so, we used images with regions that exhibit heterogeneous characteristics in spatial terms. Specifically, in Figure 8, we see images featuring objects with different spatial characteristics. These images all have two distinct regions. Firstly, Figure 8a is a spatially coherent image. Both distinct regions are strongly defined by their positions. Secondly, Figure 8b is partially spatially coherent. The region in pink is coherent, but the green background is not. Thirdly, Figure 8c is a spatially incoherent image. By using the results in Figure 8a–c, we intend to illustrate the impact of a sensible adjustment of α:

(i) In Image A, a low value of α (e.g., α=0.60) increases the importance of the spatial information during the image smoothing, compensating for the color blending that exists in the outer limits of the pink region. Note that very low values of α (α∈[0.00,0.30]) give excessive relevance to the positions within the image and can lead to a tonal-blind interpretation of the image, rendering clustering pixels according to their position.
(ii) In Image B, an intermediate value of α (e.g., α=0.80) leads to greater relevance of the color information while still considering the spatial information. The reason is that spatial information is relevant to clustering the (spatially-coherent) pink region.
(iii) In Image C, a high value of α (e.g., α=1.00) makes CAS-SFCMs fully reliant on the tonal information. Otherwise said, we expect the pixels in the image to be clustered according to their color, not according to the positions they take in the image. The pink region in the image shall be clustered together, although it is spatially dispersed.

Note that α can positively affect the smoothing when adequately set, as seen in Figure 8b with α=0.80. In addition, α can negatively affect the smoothing if wrongly employed, as observed in Figure 8c with α=0.60.

## 5. Quantitative Validation of CAS-SFCMs

CAS-SFCMs has two main objectives in terms of image smoothing. The first one is intra-region color regularization, and this shall materialize in individual regions acquiring homogeneous tones as the number of iterations progresses. The second one is inter-region difference preservation; this shall materialize as distinct regions not blending with each other as the image smoothing progresses. While the goals might not be fully compatible (see [53]), both shall be accounted for when evaluating a method for CAS. This section is devoted to qualitatively and quantitatively analyzing the intra-region color regularization and the inter-region difference preservation of CAS-SFCMs. In order to do so, we present an image dataset of real-world images in Section 5.1. These images were subjected to CAS, with the smooth images analyzed using the local homogeneity measures discussed in Section 5.2. Lastly, Section 5.3 presents the results in visual and quantitative terms.

### 5.1. Image Dataset

**Figure 9:**
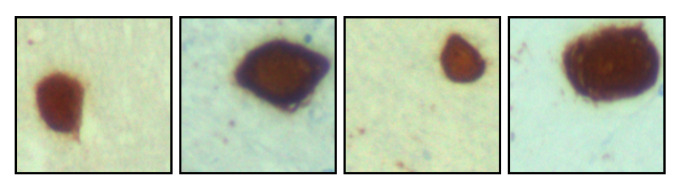
Image patches of neural tissue extracted from patients affected by Progressive Supranuclear Palsy. The darker objects represent deposits of tau protein, highlighted by the effect of immuno-histochemistry.

For the present experimental setup, we selected neural tissue images used in the research on neurodegenerative diseases. Specifically, the images are from immununo-histochemical tau-stained neural tissues from patients who had Progressive Supranuclear Palsy (PSP, [54,55]). We selected some patches, as shown in Figure 9, featuring stained tau protein deposits, the study of which is relevant to understanding the nature and evolution of PSP [56,57]. We selected these images because the immunostaining procedure generates gradual color transitions and unstructured textures in the background, which is a paradigmatic case of application for CAS. Hence, an image smoothing method capable of homogenizing the brown tones of tau protein without blending it with other image regions can be useful to automate image analysis.

### 5.2. Quantification Strategy

Quality evaluation of a smoothing process is far from evident. There is no clear definition of the goal of image smoothing for CAS or CUS. Soft definitions can be used, referring to signal regularisation or defect/artifact removal. However, there is usually no ground truth or even a strict definition that might yield mathematical expressions unless creating specific scenarios in which images are distorted prior to CAS [58]. Some image processing tasks have managed to establish quantitative criteria for image quality evaluation even in such conditions. Notably, BRISQUE [59] is a no-reference evaluation method for visual quality, but it actually relies on pre-defined tonal distributions over local image patches. While NIQE [60] is free from such assumptions and has actually been used for CAS quality evaluation [61], it focuses on visual detail enhancement, which is arguably not the goal in CAS.

In CAS, the original images need to be distorted to maximize the visibility of objects and distinct regions. Such maximization needs to be coupled with the removal of non-relevant objects from the literature. Hence, it is necessary, in our opinion, to understand the exact goal of CAS in each image. In this section, we propose a strategy to perform a quantitative analysis of image smoothing algorithms. We intended to capitalize on the idea that tones in the image should be regularized (inside objects) and kept discriminable (at object boundaries). Hence, our quantification strategy needed a local measure of homogeneity/heterogeneity, which would enable the local analysis of the tonal variation at both inter-region and intra-region pixels.

Local homogeneity [62] is a fuzzy, patch-based operator in image processing that provides a gradual measurement of the local variation at each pixel in an image. We used it to produce homogeneity maps of the tested images at each iteration of the CAS. This leads to two different interpretations. Firstly, by visually representing the homogeneity maps, we can qualitatively inspect that boundary regions keep or decrease their local homogeneity levels as iterations proceed. The opposite effect can ideally be observed in intra-region pixels. Secondly, by determining the areas of the image in which the objects’ boundaries occur, we can quantify the average homogeneity over boundary and non-boundary regions in the image.

As introduced in [62], local homogeneity for a normalized grayscale image *I* is represented as a map $H_{I}:\Omega \mapsto \left[0,1\right]$ given by

(4) $$H_{I}\left(x,y\right)=1-\overset{\begin{array}{c} i=n\\ j=n \end{array}}{\underset{\begin{array}{c} i=-n\\ j=-n \end{array}}{⋁}}I\left(x+i,y+j\right)+\overset{\begin{array}{c} i=n\\ j=n \end{array}}{\underset{\begin{array}{c} i=-n\\ j=-n \end{array}}{⋀}}I\left(x+i,y+j\right)\;,$$

where n∈N is a parameter controlling the scale. Note that, as it is customary in convolution or patch-based analysis, any (x+i,y+j)∉Ω is ignored.

The definition in Equation (4) can be adapted to multichannel images as long as the channel information is represented in [0,1]. Let *I* be a *k*-channel image. Its local homogeneity map $H_{I}^{*}$ is computed as

(5) $$H_{I}^{*}\left(x,y\right)=\frac{1}{k}\sum_{i=1}^{k}H_{I_{i}}\left(x,y\right)\;,$$

with $I_{i}$ representing the *i*-th channel in image *I*.

In this work, we use $H^{*}$ to measure the homogeneity in CIELab-encoded images. Note that this measure is not intended to be a quality metric for CAS. It does perform a numerical interpretation of the process and sheds light on how the process develops, but it should not serve as a standalone comparison of CAS algorithms. Otherwise said, it serves the purpose of understanding the behaviour of our algorithm, especially in terms of convergence, but should be carefully used if intending to produce meaningful, comparable quantifications of the performance of different algorithms. In fact, there is no widely accepted quality metric or function for such a goal.

### 5.3. Experimental Results

In this experiment, we intended to analyze, both qualitatively and quantitatively, whether CAS-SFCMs effectively produces intra-region regularization and, concurrently, inter-region boundary preservation. In order to do so, we applied CAS-SFCMs to the images in Figure 9, using α=0.5 and setting the number of clusters to r=2. These images are good representatives of a real problem because, although the immunostaining highlights the tau protein with a characteristic brownish tone, it is also spread over the neural tissue, making the image rugged and hectically textured. Moreover, the physical nature of the process makes the deposit boundaries gradually merge with the background, hence raising the need for inter-region boundary preservation (avoiding, e.g., blurring or staircasing effects [53].

**Figure 10:**
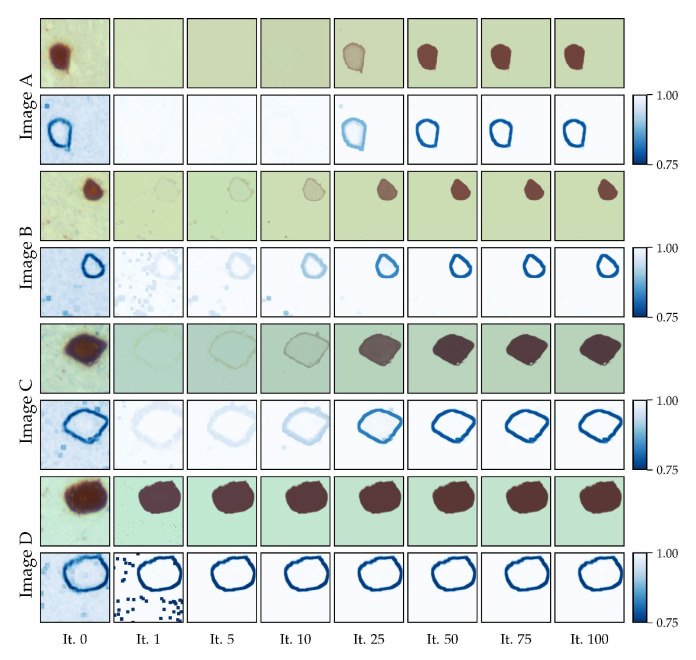
Different stages of the smoothing of the images in Figure 9 using CAS-SFCM. The experiment was run using two clusters and α=0.5. Each image is displayed for different iterations (It.), together with the local heterogeneity map computed as in Section 5.2.

**Figure 11:**
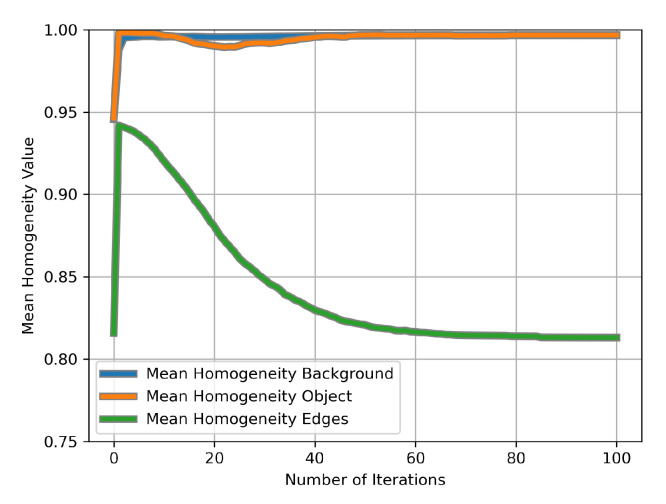
Average homogeneity in different regions of the images in Figure 9 for different numbers of iterations. The edges are defined as a seven-pixel-wide band at the boundaries of the tau protein deposits.

In Figure 10, we present the results after a different number of iterations of CAS-SFCMs applied to the images in Figure 9, together with their homogeneity maps computed as depicted in Section 5.2. Note that the left-most column in the figure contains the original images (and their local homogeneity map). Additionally, Figure 11 contains the average local homogeneity for the (a) background (b) tau deposits and (c) a seven-pixel-wide boundary region.

In Figure 10, we observe that CAS-SFCMs actually achieves its dual goal, as can be seen from both the color representation and the local homogeneity map. In fact, the most relevant fact is that early iterations of the algorithm actually perform poorly. This is due to the random nature of the Forgy initialization, which provides no guarantee of good correspondence between the centroids and the distinct regions in the image. While other alternatives for CAS that do not suffer from this slow start (such as Anisotropic Diffusion, in any of its many versions [13]), it is automatically solved in CAS-SFCMs after some iterations. Nevertheless, it is to be taken into account when we intend to execute a low number of iterations. It is noteworthy that both objects and background are, tonally, quasi-flat, leading to a local homogeneity close to 1. Figure 11 provides a quantitative representation of the facts observed in Figure 10. In early iterations, CAS-SFCMs seems to have a pernicious effect, especially in terms of homogeneity at boundary regions. However, this is related to the random selection of centroids and is solved along the iterations in the clustering process. It is also noteworthy that the process of intra-object tonal regularization is not fully monotonic, and in Figure 11, we can even observe how it takes a certain number of iterations to adjust the centroids to the actual distribution of the pixels in the spatio-tonal universe.

CAS-SFCMs is very well-suited for CAS of images with 2D shapes to be preserved. While hardly affected by textures, CAS-SFCMs can potentially produce misleading results in situations where 1D-like structures (as lines, circles, or tree-like delineations) are to be preserved. Examples of these structures can be found, e.g., in retinal vessel segmentation [63,64]. While these visual artifacts are absent from the specific application this work focuses on, we recommend that researchers reduce the relevance of the spatial comparison when applying CAS-SFCMs to images containing them or to use CAS algorithms based on local information (such as Anisotropic Diffusion [12,13] or bilateral filtering [14]).

## 6. Conclusions

In this paper, we propose CAS-SFCMs, a content-aware image smoothing strategy that is inspired by fuzzy clustering and uses spatial information from regions within an image. This strategy includes a spatio-tonal parameter that enables better performance in scenarios where the images possess specific spatial coherences. We have shown that CAS-SFCMs is inherently resistant to the initial selection of the cluster centroids, converging to nearly identical results regardless of the initial cluster centroids. It also achieves intra-region color regularization and avoids inter-region edge blurring, removing textures from within the regions and sharpening the boundaries of regions without damaging their shapes. In terms of the configuration and adaptation of different scenarios, CAS-SFCMs is robust in terms of the number of centroids and random initialization, but it also allows for the selection of metrics for spatial and tonal distance calculations, enabling adaptation in different scenarios.

As future lines of work, we intend to fully integrate CAS into visual inspection tools for neural tissue analysis. Our intention is to evaluate and potentially predict the level of smoothness that represents maximum comfort for experts in their medical practice. Moreover, we intend to use this information to automatically determine the number of clusters on the basis of the spatio-tonal distribution of the original image.

## Data Availability

Data and source code is available upon contact with the corresponding author.
